# Supplementary material for: Characterizing transition-metal dichalcogenide thin-films using hyperspectral imaging and machine learning
Source: Sci Rep. 2020 Jul 14;10:11602. doi: 10.1038/s41598-020-68321-7 (PMC7360754; doi:10.1038/s41598-020-68321-7)
Supplement: Supplementary file 1 — Supplementary information [file 41598_2020_68321_MOESM1_ESM.docx]

**Characterizing Transition-Metal Dichalcogenide Thin-Films using Hyperspectral Imaging and Machine Learning**

Brian Shevitski1,2,3,4, Christopher T. Chen4, Christoph Kastl4,5, Tevye Kuykendall4, Adam Schwartzberg4, Shaul Aloni4* and Alex Zettl1,2,3*

^1^Department of Physics, University of California at Berkeley, Berkeley, CA 94720, U.S.A.

^2^Materials Sciences Division, Lawrence Berkeley National Laboratory, Berkeley, CA 94720, U.S.A.

^3^Kavli NanoEnergy Sciences Institute at the University of California at Berkeley and the Lawrence Berkeley National Laboratory, Berkeley, CA 94729, U.S.A.

^4^The Molecular Foundry, Lawrence Berkeley National Laboratory, Berkeley, CA 94720, U.S.A.

^5^Walter-Schottky-Institute and Physik Department, Technical University of Munich, Garching, 85748, Germany

*To whom correspondence should be addressed: azettl@berkeley.edu, saloni@lbl.gov

**Figure SI1.** Photoluminescence and Raman characterization of WS_2_ thin films and single crystals. Similar to bulk WS_2_, multilayer films (2 nm nominal thickness) exhibit no photoluminescence, as shown in (a). Monolayer films (0.8 nm nominal thickness) and monolayer single crystals (1-2 $\mu$m lateral sized triangles) have distinct PL and Raman peaks as shown in (b) and (c), respectively.

**Figure SI2.** Runtimes of the primary computational bottlenecks of both MVA and traditional analysis for the 4DSTEM data set presented in the text. PCA (a) and ICA (b) decomposition into a basis with less than ~3000 components is faster than traditional template matching 4DSTEM analysis (a). All tests were performed using the CPU on the same laptop computer (Macbook pro, 2.3 GHz 8-core Intel i9). Template matching was accomplished using the match_template method in scikit-image, which utilizes fast cross-correlation and identifies peaks in correlation image. Data pre-treatment is identical for both methods and determination of grain sizes is orders of magnitude faster for both methods so the computational costs of these steps is not presented here.

**Figure SI3.** Number of ICA signal components as a function of ICA output dimension as determined by the workflow described in the text. The dashed line shows the number of ICA components (49) chosen for the grain size analysis.

**Figure SI4.** First 120 k-space PCA decomposition factors for the 4DSTEM data set presented in the manuscript.

**Figure SI5.** First 120 real space PCA decomposition loadings for the 4DSTEM data set presented in the manuscript.
